# Impact of Polypharmacy on the Rehabilitation Outcome of Japanese Stroke Patients in the Convalescent Rehabilitation Ward

**DOI:** 10.1155/2016/7957825

**Published:** 2016-11-29

**Authors:** Eiji Kose, Riku Maruyama, Susumu Okazoe, Hiroyuki Hayashi

**Affiliations:** ^1^Department of Pharmacotherapy, School of Pharmacy, Nihon University, Chiba, Japan; ^2^Department of Pharmacy, Sagami Rehabilitation Hospital, Kanagawa, Japan

## Abstract

*Background*. A risk factor associated with stroke onset is chronic kidney disease (CKD). To prevent stroke reoccurrence, it is necessary to strictly manage blood pressure, lipids, and plasma glucose. Therefore, some cases are forced to polypharmacy, elderly patients in particular. Polypharmacy often leads to adverse drug reactions and has the potential to negatively affect the rehabilitation of stroke patients. The aim of the present study was to investigate the effects of polypharmacy using a functional independence measure (FIM).* Methods*. A total of 144 stroke patients with CKD were included in the present analysis. We divided stroke patients into those taking six or more drugs (polypharmacy group) and those taking less than six drugs (nonpolypharmacy group) upon admission. Patient background features, laboratory data, and FIM scores were compared.* Results*. FIM-Motor (FIM-M) efficiency, age, and diabetes mellitus were positively associated with polypharmacy. FIM-M efficiency in the polypharmacy group was significantly lower than in the nonpolypharmacy group.* Conclusion*. Polypharmacy interferes with the effect of rehabilitation in stroke patients with CKD. Pharmacists and doctors should make efforts to optimize medications to be able to respond to the outcome of each patient.

## 1. Introduction

Stroke is the fourth leading cause of death in Japan and ranks number one in terms of required nursing care. While the number of deaths due to a stroke has been decreasing, according to a study group of the Ministry of Health, Labour, and Welfare, the prevalence of stroke and associated nursing care will continue to increase until 2025. Even if appropriate treatment after stroke onset increases patient survival, there are cases in which some neurological disorders such as allopathy or motility disorder and high-order function disorders such as aphasia or memory impairment remain. In such cases, the role of the convalescent rehabilitation ward is to lead patients into social rehabilitation by improving their activities of daily living (ADL). There is an expectation that the demand for rehabilitation will continue to grow as the prevalence of strokes continues to increase.

One risk factor associated with stroke onset is chronic kidney disease (CKD), according to the 2015 Japanese guidelines for the management of strokes and national and international epidemiological studies [1–5]. Furthermore, the Fukuoka Stroke Registry reported that 32.1% of patients hospitalized for a stroke also had CKD [6]. In Japan, 30% of men and 40% of women aged over 65 years are CKD patients [7]. The number of CKD patients in Japan is approximately 13,300,000 and continues to increase [8]. Lifestyle-related diseases such as hypertension, dyslipidemia, and diabetes mellitus have been implicated in the development and progression of CKD. CKD management is extremely important, because it is a risk factor not only for end-stage renal failure, but also for coronary artery diseases such as myocardial infarction or stroke [2, 9].

For CKD patients, strict management of their blood pressure, lipids, and plasma glucose is required in order to prevent a stroke reoccurrence [1]. As a consequence, some patients are forced to polypharmacy. Elderly patients often experience adverse drug reactions due to polypharmacy because their decrease in organ function is associated with the metabolism of multiple drugs [10, 11]. Therefore, we consider that there is a possibility that polypharmacy may negatively affect the rehabilitation outcome of stroke patients in the convalescent rehabilitation wards. Previous studies have reported the intervention of a nutritional support team or stroke subtype associated with the functional independence measure (FIM), which is an evaluation index of ADL [12, 13]. However, few reports exist on the influence of drugs on FIM. Therefore, we investigated the effects of drugs on FIM in stroke patients with CKD.

## 2. Materials and Methods

### 2.1. Subjects

Among the 244 stroke patients with CKD who were discharged from Sagami Rehabilitation Hospital between January 2013 and July 2014, 144 patients were included in the present study. The frequency of adverse drug reactions rapidly increases when six or more drugs are prescribed to elderly people aged over 65 years [14]. Therefore, it is desirable to reduce the number of drugs to five or less in order to reduce the likelihood of adverse drug reactions. Thus, we defined a combination of at least six or more drugs as polypharmacy. We divided stroke patients into those taking six or more drugs (polypharmacy group) and those taking less than six drugs (nonpolypharmacy group) upon admission. In addition, CKD stage was classified according to the Japanese Society of Nephrology Evidence-based Clinical Practice Guideline for CKD 2013.

### 2.2. Investigation Items

We examined the following parameters to elucidate any differences between the polypharmacy and nonpolypharmacy groups: patient gender, age, body mass index, days from stroke onset to admission, length of stay, number of drugs upon admission, number of complications, medical history (e.g., hypertension, diabetes mellitus, dyslipidemia, cardiovascular disease, dementia, and epilepsy), FIM score on admission and at discharge [total (FIM-T), motor (FIM-M), and cognitive (FIM-C) items], and FIM efficiency. Laboratory data included albumin (Alb), serum creatinine (Scr), creatinine clearance (Ccr), estimated glomerular filtration rate (eGFR), glycosylated hemoglobin (HbA1c), fasting plasma glucose level (FPG), total cholesterol (T-Cho), total lymphocyte count (TLC), and white blood cell (WBC) count. We assessed these data upon admission to a convalescent rehabilitation ward. Drug data included the use of antihypertensive, antidiabetic, antihyperlipidemic, bisphosphonate, antidementia, antiepileptic, antianxiety, and hypnotic sedative drugs. Accordingly, these data were included as well. In addition, in many countries, including the United States, Scr had been measured using the Jaffe method. However, Scr is measured using an enzymatic method in Japan. The Modification of Diet in Renal Disease (MDRD) equation based on Scr measured using the enzymatic method was published in 2006. In consideration of racial differences, the Japanese coefficient of 0.741 has been published by the Japan Society of Nephrology.

*Male*

(1) $$\begin{array}{c} \text{GFR}=0.741\times 175\times {\left(\;\text{Age}\;\right)}^{-0.203}\times {\left(\;\text{Scr}\;\right)}^{-1.154} \end{array}$$

*Female*

(2) $$\begin{array}{c} \text{GFR}=0.741\times 0.742\times 175\times {\left(\;\text{Age}\;\right)}^{-0.203}\times {\left(\;\text{Scr}\;\right)}^{-1.154} \end{array}$$

In the present study, eGFR was calculated based on the MDRD equation as shown above.

### 2.3. Outcome Measure

The primary outcome of the present study is FIM-M efficiency. We compared the FIM-M efficiency between the polypharmacy and nonpolypharmacy groups to investigate the effect of drug number on FIM-M efficiency. ADL indicators such as the FIM and the Barthel index were used in the evaluation during the recovery period [15]. Given that the reliability of FIM has been previously confirmed [16], we used FIM to evaluate ADL in the present study. FIM is a scale for assessing disability based on five items associated with cognition and 13 items associated with motor function in daily life, with each item scoring from one (requiring maximum assistance) to seven (full independence). Thus, the highest possible total score is 126 and the lowest is 18, with higher scores indicating greater autonomy. FIM efficiency shows the improvement of rehabilitation per day. The FIM efficiency score was calculated as the FIM score at discharge minus the FIM score upon admission/length of stay.

The rehabilitation team in the convalescent rehabilitation ward of the Sagami Rehabilitation Hospital was composed of a physician, experienced nurses, physical therapist, occupational therapist, a speech-language-hearing therapist, and a pharmacist. The rehabilitation team discussed and evaluated the patients' FIM as a team. Drugs used during the observation period were changed to generic drugs or to drugs of the same type, or there were no drug changes. The same units of rehabilitation were carried out for all patients regardless of their FIM score, stroke severity, or length of stay.

### 2.4. Data Analysis

Results are presented as the mean ± standard deviation (SD). A normality test was performed to compare the data volume between the two groups. We used a Student's *t*-test for normally distributed data or a Mann–Whitney *U*-test for data that were not normally distributed. We used *χ* ^2^ test or Fisher's exact test to compare categorical data. Next, we adjusted for confounding variables and performed a multiple logistic regression analysis with the presence or absence of polypharmacy as a dependent variable to investigate the association of polypharmacy with patient background, FIM efficiency, laboratory data, and drugs used on admission. We chose significant factors as independent variables (age, diabetes mellitus, and FIM-M efficiency) on the basis of the univariate analysis results. Confirmation was achieved by a multiple logistic regression analysis when no multicollinearity existed between factors using Pearson or Spearman's rank-correlation coefficients. Results were considered significant at *p* < 0.05. All statistical analysis was performed using JMP® Pro (version 12, SAS Institute Inc., Cary, NC, USA).

### 2.5. Ethics Regulation

The present study was conducted with the approval of the Sagami Rehabilitation Hospital ethics committee. In addition, the present study was conducted with the approval of the School of Pharmacy, Nihon University Ethics Committee. This was a retrospective study using medical records, which complied with the Declaration of Helsinki and the “Ethical Guidelines for Clinical Research.”

## 3. Results

### 3.1. Selection of Subjects

**Figure 1:**
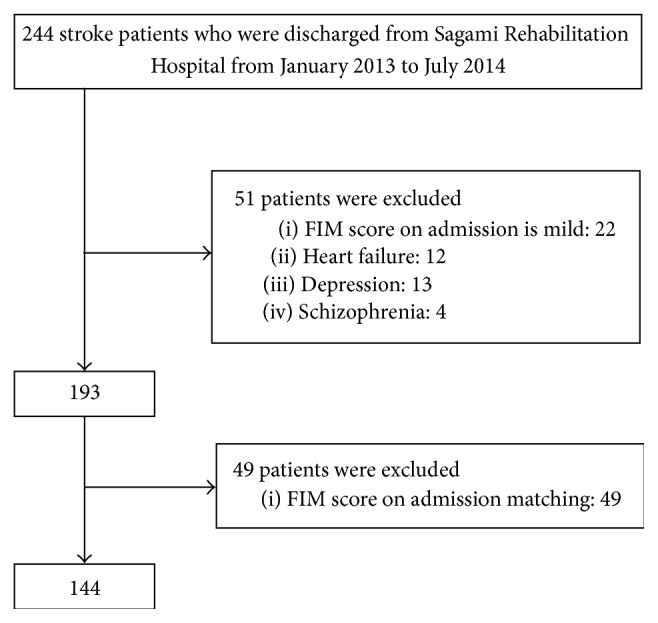
Flow chart of the subject selection process.

Figure 1 shows a flow chart of the selection of subjects who were discharged from the Sagami Rehabilitation Hospital between January 2013 and July 2014. According to a previous study [17], we defined an FIM-T of 110–126 points as mild, 80–109 points as moderate, and 18–79 points as severe. We excluded 22 patients with a mild FIM-T score upon admission, as it was difficult to ascertain their FIM efficiency because of the ceiling effect. In addition, 12 patients were excluded from the study because they were reported as having heart failure that may limit their movement according to the degree of severity as established by the New York Heart Association. Thirteen patients with depression and four patients with schizophrenia were also excluded because they were restricted in rehabilitation and their FIM could not improve because they were unable to complete routine rehabilitation [18, 19]. We also excluded 49 patients with matching FIM scores upon admission. Finally, a total of 144 patients were selected for the present study, 48 in the polypharmacy group and 96 in the nonpolypharmacy group. Furthermore, we surveyed patients with a CKD stage of 1–4.

### 3.2. Comparison of Patient Background Characteristic Data

Patient background characteristic data for the polypharmacy and nonpolypharmacy groups are given in Table 1. The number of females was significantly higher in the polypharmacy group compared with that in the nonpolypharmacy group (60.4% versus 42.7%, *p* = 0.045). Similarly, the number of drugs upon admission, number of complications, and a medical history of diabetes mellitus and dementia were significantly higher in the polypharmacy group compared with those in the nonpolypharmacy group (drugs upon admission: 7.8 ± 2.2 versus 3.4 ± 1.2, *p* ≤ 0.0001; number of complications: 3.8 ± 2.1 versus 2.7 ± 1.8, *p* = 0.001; diabetes mellitus: 41.7% versus 20.8%, *p* = 0.008; dementia: 25.0% versus 8.3%, *p* = 0.006). On the other hand, age, days from stroke onset to admission, length of stay, and a medical history of hypertension, dyslipidemia, cardiovascular disease, or epilepsy were not significantly different between the two groups.

### 3.3. Comparison of FIM Score upon Admission and at the Time of Discharge and FIM Efficiency

The FIM score upon admission and at the time of discharge and the FIM efficiency between the polypharmacy and nonpolypharmacy groups are given in Table 2. In the polypharmacy group, the FIM-T score at discharge was significantly lower compared with that in the nonpolypharmacy group (81.0 ± 29.6 points versus 90.5 ± 25.5 points, *p* = 0.047). Similarly, the FIM-C at discharge, FIM-T, and FIM-M efficiency in the polypharmacy group were significantly lower compared with those in the nonpolypharmacy group (FIM-C at discharge: 25.0 ± 7.7 points versus 28.1 ± 6.4 points, *p* = 0.011; FIM-T efficiency: 0.15 ± 0.14 versus 0.23 ± 0.17, *p* = 0.007; FIM-M efficiency: 0.13 ± 0.12 versus 0.20 ± 0.15, *p* = 0.009). In contrast, the FIM-T, FIM-M, and FIM-C upon admission, FIM-M at discharge, and FIM-C efficiency were not significantly different between the two groups.

### 3.4. Comparison of Laboratory Data

The laboratory data between the polypharmacy and nonpolypharmacy groups are given in Table 3. The FPG in the polypharmacy group was significantly higher compared with that in the nonpolypharmacy group (134.7 ± 39.0 mg/dL versus 119.8 ± 38.1 mg/dL, *p* = 0.029). On the other hand, the Alb, Scr, Ccr, eGFR, HbA1c, T-Cho, TLC, and WBC were not significantly different between the two groups.

### 3.5. Comparison of Oral Drugs Being Taken upon Admission

The number and type of drugs being taken upon admission between the polypharmacy and nonpolypharmacy groups are given in Table 4. The number of patients on antidiabetic drugs in the polypharmacy group was significantly higher compared with that in the nonpolypharmacy group (33.3% versus 9.4%, *p* = 0.0003). Similarly, the number of patients on antidementia and antianxiety drugs in the polypharmacy group was significantly higher compared with that in the nonpolypharmacy group (antidementia drug: 12.5% versus 2.1%, *p* = 0.01; antianxiety drug: 10.4% versus 0%, *p* = 0.001). On the other hand, the number of patients on antihypertensive, antihyperlipidemic, bisphosphonate, antiepileptic, and hypnotic sedative drugs was not significantly different between the two groups.

### 3.6. Multiple Logistic Regression Analysis

We evaluated all 144 patients using a multiple logistic regression analysis. Various factors associated with polypharmacy were used in the analysis. Significant differences were observed in FIM-M efficiency, age, and the presence of diabetes mellitus (Table 5).

### 3.7. Effect of Polypharmacy on FIM-M Efficiency

**Figure 2:**
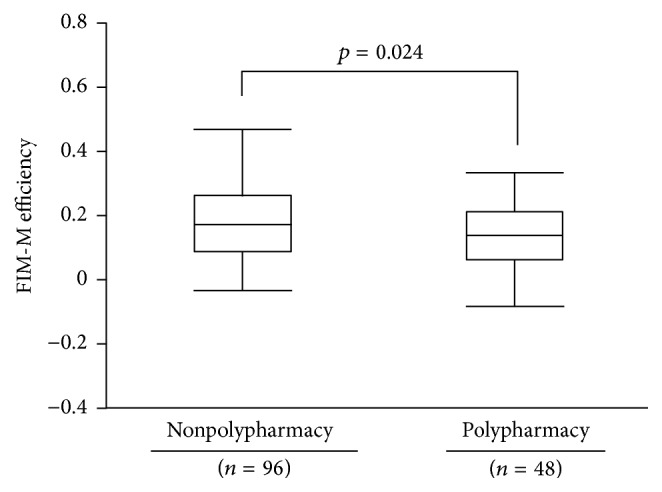
Effect of polypharmacy on FIM-M efficiency. We used the Mann–Whitney *U*-test to compare FIM-M efficiency between polypharmacy and nonpolypharmacy. Polypharmacy: six or more drugs; nonpolypharmacy: less than six drugs.

We examined the effect of the presence or absence of polypharmacy on FIM-M efficiency. FIM-M efficiency was significantly lower in the polypharmacy group compared with the nonpolypharmacy group (0.13 ± 0.12 versus 0.20 ± 0.15, *p* = 0.024) (Figure 2).

## 4. Discussion

The most important finding of the present study was the positive association of polypharmacy with FIM-M efficiency, age, and diabetes mellitus. In addition, we revealed that when it comes to polypharmacy, it is difficult to obtain FIM-M efficiency when compared with nonpolypharmacy.

It is important to strictly manage blood pressure, lipids, and plasma glucose as a secondary preventive measure against stroke [1]. CKD is one risk factor of a stroke and its onset and progression is associated with lifestyle-related diseases such as hypertension, dyslipidemia, and diabetes mellitus [1]. Therefore, it is often the case that stroke patients with CKD are necessarily associated with polypharmacy. Adverse drug reactions or the risk of adverse drug interactions caused by polypharmacy has been reported globally and has become a major obstacle in the safe and reliable treatment of a stroke [20]. In particular in the convalescent rehabilitation ward, we cannot deny the possibility that the expression of adverse drug reactions affects FIM. Therefore, we examined the effect of polypharmacy on FIM.

We revealed that diabetes mellitus was the most common independent risk factor with approximately 4.1-fold increased risk of polypharmacy. The number of drugs used by patients with diabetes mellitus was approximately two agents larger than that used by patients without diabetes mellitus (6.3 ± 2.7 versus 4.4 ± 2.4, *p* < 0.0001). Even if the control of plasma glucose is improved with a monotherapy of an antidiabetic drug, sometimes the control deteriorates and the use of a multidrug combination is necessary. In addition, it is known that plasma glucose control deteriorates due to comorbidity or concomitant drugs. Oishi et al. [21] reported a change in the antidiabetic drugs prescribed from 2002 to 2011. While monotherapy accounted for 52.8% of the cases in 2002, it had significantly decreased to 35.9% by 2011. Subsequently, a combination therapy increased over time and a dual therapy was the most common treatment protocol by 2011, accounting for 33.3% of cases. A triple therapy accounted for 24.8% of cases, although this treatment protocol has seen a twofold increase compared to 2002. A quad combination therapy increased 14.8-fold from 2002 to 2011. Thus, combination therapies have the potential to be further increased because of the sodium-glucose transporter 2 inhibitors that are currently on the market. We believe that the trends of antidiabetic drugs are reflected as the factor of polypharmacy in patients with diabetes mellitus.

In the present study we revealed the possibility that the FIM-M efficiency was also associated with polypharmacy. In other words, the FIM-M efficiency with six or more drugs was significantly lower compared with less than six drugs. This finding suggested that, in regard to polypharmacy, it was difficult to obtain FIM-M efficiency. Thus, we consider it desirable to reduce the number of prescribed drugs to five or fewer drugs when possible.

The results of the univariate analysis demonstrated that the proportion of antidiabetic, antidementia, and antianxiety drugs in the polypharmacy group was significantly higher compared with those in the nonpolypharmacy group. Therefore, these drugs might be associated with FIM-M efficiency. In the relationship between antidiabetic drugs and FIM-M efficiency, maximum oxygen uptake of diabetic patients without an insulin treatment was 20% less than in healthy people [22]. In addition, a decrease in the muscle mass of the lower limbs or in muscle strength of elderly patients with type 2 diabetes mellitus was significantly greater compared with nondiabetic elderly patients [23]. Furthermore, in a previous study we revealed that when using sulfonylurea (SU) drugs, it was difficult to obtain FIM efficiency compared with using *α*-glucosidase inhibitor (*α*-GI) drugs [24]. Thus, we consider that it is difficult to obtain FIM efficiency because of diabetic patients' reduced ability to exercise, likely due to an insufficient supply of ATP caused by a failure of oxidative phosphorylation in their mitochondria [25]. In other words, as the average age of patients in the polypharmacy group was 74 years, the energy production required in order to exercise would have been decreased by a reduced incorporation of plasma glucose into the periphery due to their decrease in skeletal muscle. Therefore, we should further examine whether reinforcing muscular strength by resistance training or the administration of branched-chain amino acids would improve muscle-building or physical strength for patients who have a decreased muscle mass [26–28].

The reason why the use of antidementia drugs was significantly higher in the polypharmacy group was as follows. Dementia is a chronic disease that increases in prevalence as one ages, and there are some cases in which patients with dementia have a chronic disease in addition to dementia because they are elderly. Furthermore, in recent years, drugs with different mechanisms of action, such as memantine, are on the market and the usefulness of administering cholinesterase inhibitors in combination with memantine has been demonstrated in patients with moderate to severe Alzheimer's disease. Therefore, patients with these diseases have an especially high risk of polypharmacy. In the present study, the number of drugs upon admission in patients with Alzheimer's disease tends to be higher compared with patients without Alzheimer's disease (5.8 ± 2.8 versus 4.8 ± 2.6, *p* = 0.070). Alzheimer's disease often presents as recent memory impairment and behavioral psychological symptoms of dementia such as wandering, restlessness, and emotional instability. In Lewy body dementia, mental symptoms such as delusion and hallucinations are frequent. There are some cases where antipsychotic, antianxiety, and psychotropic drugs are administrated as supportive care for these symptoms. Thus, this factor has the potential to promote polypharmacy.

Associated with antidementia drugs and FIM-M efficiency are some cases in which patients with Alzheimer's disease forget to eat or do not feel hungry due to cognitive dysfunction. In addition, as the dementia progresses it is difficult to eat because of emotional instability resulting in malnutrition which caused the anorexia and weight loss [29]. Soysal et al. [30] reported that cholinesterase inhibitors were associated with weight loss and elderly patients with dementia often have protein energy malnutrition [31]. Therefore, we believe that it is difficult to obtain FIM-M efficiency because dementia patients are prevented from recovering their body's functions due to less motivation, decreased strength, and fatigue due to malnutrition. We consider it important to perform nutritional management in order to improve strength and motivation that will enhance the effects of rehabilitation and consequently improve motor function.

In the present study we identified that age is also likely to be a factor of polypharmacy with a risk of up to 1.04-fold imposed by age. Toba et al. [10] reported that the number of drugs taken increased with age, a finding supported in the present study.

The present study contains several limitations. First, it was a cross-sectional, single-center study with only a small number of patients undergoing analysis. Second, we hypothesized that side effects caused by renal dysfunction may be involved in rehabilitation outcome. However, there were no significant differences in renal function between polypharmacy group and nonpolypharmacy group. Thus, in present study, we were unable to confirm the association between polypharmacy and renal function. Third, the severity of the dementia was unclear. In general, the Hasegawa Dementia Scale-Revised (HDS-R) or Mini-Mental State Examination (MMSE) is used as an objective assessment scale for the severity of dementia. However, in the present study we could not analyze these data adequately and consider this factor to likely affect our results. However, we do believe that this influence on the results was small because the FIM-C scores on admission were not significantly different between the polypharmacy and nonpolypharmacy groups. Fourth, doses and types of drugs were not considered. We consider that this factor was likely to have affected the results of the present study because, especially for antidiabetic drugs, the frequency of hypoglycemia was different depending on the dose and type of drugs used. Thus, in the future, it is necessary to verify these points by a prospective cohort study.

## 5. Conclusion

Polypharmacy interferes with the rehabilitation of stroke patients with CKD. Pharmacists should make efforts to optimize prescriptions to be able to respond to the outcome of each individual patient, bearing in mind that each prescribed drug is necessary for the patient.

## Figures and Tables

**Table 1:** Comparison of patient background characteristic data.

Characteristic	All patients (*n* = 144)	Polypharmacy group (*n* = 48)	Nonpolypharmacy group (*n* = 96)	*p* value
Gender, females *n*, (%)	70 (48.6)	29 (60.4)	41 (42.7)	0.0450
Age (y)	70.9 ± 10.2	72.7 ± 10.1	70.0 ± 10.2	0.1475
Body mass index (kg/m^2^)	21.4 ± 3.8	21.8 ± 3.8	21.2 ± 3.8	0.3895
Days from stroke onset to admission (d)	44.3 ± 25.7	46.0 ± 18.0	43.1 ± 28.7	0.4542
Length of stay (d)	123.9 ± 46.6	123.7 ± 45.2	123.9 ± 47.5	0.9789
Number of drugs upon admission	4.9 ± 2.7	7.8 ± 2.2	3.4 ± 1.2	<0.0001
Number of complications	3.0 ± 2.0	3.8 ± 2.1	2.7 ± 1.8	0.0011
Medical history *n*, (%)				
Hypertension	119 (82.6)	40 (83.3)	79 (82.3)	0.8764
Diabetes mellitus	40 (27.8)	20 (41.7)	20 (20.8)	0.0085
Dyslipidemia	36 (25.0)	15 (31.2)	21 (21.9)	0.2207
Cardiovascular disease	40 (27.8)	15 (31.3)	25 (26.0)	0.5107
Dementia	20 (13.9)	12 (25.0)	8 (8.3)	0.0064
Epilepsy	16 (11.1)	7 (14.6)	9 (9.4)	0.3485

Mean ± SD.

**Table 2:** Comparison of FIM score upon admission and at the time of discharge and FIM efficiency.

Characteristic	All patients (*n* = 144)	Polypharmacy group (*n* = 48)	Nonpolypharmacy group (*n* = 96)	*p* value
FIM score on admission (points)				
Total	63.5 ± 23.4	61.7 ± 22.1	64.4 ± 24.1	0.5167
Motor	40.2 ± 18.0	39.7 ± 17.1	40.4 ± 18.5	0.8346
Cognitive	23.4 ± 7.6	22.0 ± 7.3	24.1 ± 7.8	0.1276
FIM score at discharge (points)				
Total	87.4 ± 27.2	81.0 ± 29.6	90.5 ± 25.5	0.0471
Motor	60.4 ± 21.6	56.2 ± 22.9	62.4 ± 20.7	0.1017
Cognitive	27.1 ± 6.9	25.0 ± 7.7	28.1 ± 6.4	0.0119
FIM efficiency (points/d)				
Total	0.21 ± 0.16	0.15 ± 0.14	0.23 ± 0.17	0.0070
Motor	0.18 ± 0.14	0.13 ± 0.12	0.20 ± 0.15	0.0095
Cognitive	0.03 ± 0.05	0.02 ± 0.04	0.03 ± 0.05	0.1590

Mean ± SD.

**Table 3:** Comparison of laboratory data.

Characteristic	All patients (*n* = 144)	Polypharmacy group (*n* = 48)	Nonpolypharmacy group (*n* = 96)	*p* value
Alb (g/dL)	3.8 ± 0.5	3.7 ± 0.5	3.8 ± 0.4	0.6261
Scr (mg/dL)	0.7 ± 0.3	0.7 ± 0.2	0.7 ± 0.3	0.8645
Ccr (mL/min)	52.0 ± 17.7	51.2 ± 19.6	52.4 ± 16.7	0.7503
eGFR (mL/min/1.73 m^2^)	78.3 ± 31.5	75.2 ± 29.2	80.4 ± 32.5	0.2622
HbA1c (NGSP) (%)	6.1 ± 1.1	6.3 ± 1.2	5.9 ± 0.9	0.0923
FPG (mg/dL)	124.8 ± 38.9	134.7 ± 39.0	119.8 ± 38.1	0.0295
T-Cho (mg/dL)	175.6 ± 35.0	173.1 ± 39.5	177.0 ± 3.3	0.5463
TLC (×10^3^/*μ*L)	1.5 ± 0.6	1.5 ± 0.5	1.5 ± 0.6	0.9251
WBC (×10^3^/*μ*m)	6.3 ± 1.9	6.3 ± 1.7	6.4 ± 1.9	0.8085

Mean ± SD.

**Table 4:** Comparison of oral drugs being taken upon admission.

Characteristic	All patients (*n* = 144)	Polypharmacy group (*n* = 48)	Nonpolypharmacy group (*n* = 96)	*p* value
Drugs *n*, (%)				
Antihypertensive	101 (70.1)	35 (72.9)	66 (68.8)	0.6065
Antidiabetic	25 (17.4)	16 (33.3)	9 (9.4)	0.0003
Antidyslipidemic	47 (32.6)	18 (37.5)	29 (30.2)	0.3790
Bisphosphonate	3 (2.1)	2 (4.2)	1 (1.0)	0.2158
Antiepileptic	16 (11.1)	8 (16.7)	8 (8.3)	0.1336
Antidementia	8 (5.6)	6 (12.5)	2 (2.1)	0.0101
Antianxiety	5 (3.5)	5 (10.4)	0 (0)	0.0013
Hypnotic sedative	14 (9.7)	5 (10.4)	9 (9.4)	0.8423

Mean ± SD.

**Table 5:** Multiple logistic regression analysis of various factors associated with polypharmacy.

Factor	Adjusted odds ratio	95% CI	*p* value
Age	1.0424	1.0025–1.0871	0.0366
FIM-M efficiency	0.0078	0.0002–0.1717	0.0015
Diabetes mellitus	4.1131	1.7872–9.8729	0.0008

95% CI: 95% confidence interval (*n* = 144).

## References

[1] The Japan Stroke Society (2015). *Japanese Guidelines for the Management of Stroke*.

[2] Katsumata T. (2014). CKD and stroke. *Nosotchu*.

[3] Ninomiya T., Kiyohara Y., Kubo M. (2005). Chronic kidney disease and cardiovascular disease in a general Japanese population: the Hisayama Study. *Kidney International*.

[4] Irie F., Iso H., Sairenchi T. (2006). The relationships of proteinuria, serum creatinine, glomerular filtration rate with cardiovascular disease mortality in Japanese general population. *Kidney International*.

[5] Kokubo Y., Nakamura S., Okamura T. (2009). Relationship between blood pressure category and incidence of stroke and myocardial infarction in an Urban Japanese population with and without chronic kidney disease: the suita study. *Stroke*.

[6] Kitazono T., Kumai Y., Sugimori H. (2009). Impact of hypertension and chronic kidney disease on acute ischemic stroke; the Fukuoka Stroke Registry. *Japanese Journal of Stroke*.

[7] Imai E., Horio M., Iseki K. (2007). Prevalence of chronic kidney disease (CKD) in the Japanese general population predicted by the MDRD equation modified by a Japanese coefficient. *Clinical and Experimental Nephrology*.

[8] Imai E., Horio M., Watanabe T. (2009). Prevalence of chronic kidney disease in the Japanese general population. *Clinical and Experimental Nephrology*.

[9] (2012). *Clinical Practice Guidebook for Diagnosis and Treatment of Chronic Kidney Disease*.

[10] Toba K., Akishita M., Mizuno Y. (1999). Adverse drug reaction in the elderly. *Japanese Journal of Geriatrics*.

[11] Akishita M., Teramoto S., Arai H. (2004). Incidence of adverse drug reactions in geriatric wards of university hospitals. *Japanese Journal of Geriatrics*.

[12] Senda J., Ito K., Hamada K., Kotake T., Kishimoto H., Sobue G. (2010). Investigation of inpatient rehabilitation outcomes in different ischemic stroke disease types : relationships with leukoaraiosis in MRI. *The Japanese Journal of Rehabilitation Medicine*.

[13] Usui W., Sonoda S., Suzuki T., Okamoto S., Higashiguchi T., Saitoh E. (2008). Validating a Nutrition Support Team's (NST) effect in convalescent stroke rehabilitation using the functional independence measure. *The Japanese Journal of Rehabilitation Medicine*.

[14] Kojima T., Akishita M., Kameyama Y. (2012). High risk of adverse drug reactions in elderly patients taking six or more drugs: analysis of inpatient database. *Geriatrics and Gerontology International*.

[15] Saeki S. (2009). The knowledge of the rehabilitation necessary for clinical pathways for the local stroke network. *Japanese Journal of Stroke*.

[16] Ottenbacher K. J., Hsu Y., Granger C. V., Fiedler R. C. (1996). The reliability of the functional independence measure: a quantitative review. *Archives of Physical Medicine and Rehabilitation*.

[17] Hirata Y. (2008). Present conditions and future issues concerning a liaison critical path for stroke patient-from the standpoint of a convalescence rehabilitation hospital. *The Journal of Japan Society for Health Care Management*.

[18] Senzaki A., Inamura M., Mamada T. (2009). Depression and dementia in elderly people. *Clinical Rehabilitation*.

[19] Senzaki A., Takagi H., Yamada H., Kamikozuru M. (2005). Rehabilitation for schizophrenic patients with spinal cord injury following suicidal jumping. *The Japanese Journal of Rehabilitation Medicine*.

[20] Ogawa Y., Sakoh M., Mihara K., Ogawa R., Echizen H. (2016). Factors influencing the number of drugs among elderly patients hospitalized in a rehabilitation ward. *Journal of Pharmaceutical Health Care and Sciences*.

[21] Oishi M., Yamazaki K., Okuguchi F., Sugimoto H., Kanatsuka A., Kashiwagi A. (2014). Changes in oral antidiabetic prescriptions and improved glycemic control during the years 2002–2011 in Japan (JDDM32). *Journal of Diabetes Investigation*.

[22] De Feyter H. M., Van den Broek N. M. A., Praet S. F. E., Nicolay K., Van Loon L. J. C., Prompers J. J. (2008). Early or advanced stage type 2 diabetes is not accompanied by in vivo skeletal muscle mitochondrial dysfunction. *European Journal of Endocrinology*.

[23] Leenders M., Verdijk L. B., van der Hoeven L. (2013). Patients with type 2 diabetes show a greater decline in muscle mass, muscle strength, and functional capacity with aging. *Journal of the American Medical Directors Association*.

[24] Kose E., Toyoshima M., Tachi T., Teramachi H., Kawakubo T., Hayashi H. (2015). Effects of antidiabetes drugs on functional independence measure on a subacute rehabilitation ward for stroke patients. *Pharmazie*.

[25] Kinugawa S., Tsutsui H. (2012). Insulin resistance and exercise capacity: effect of intramyocellular lipid. *Cardioangiology*.

[26] Manders R. J. F., Praet S. F. E., Meex R. C. R. (2006). Protein hydrolysate/leucine co-ingestion reduces the prevalence of hyperglycemia in type 2 diabetic patients. *Diabetes Care*.

[27] Katsanos C. S., Kobayashi H., Sheffield-Moore M., Aarsland A., Wolfe R. R. (2006). A high proportion of leucine is required for optimal stimulation of the rate of muscle protein synthesis by essential amino acids in the elderly. *American Journal of Physiology-Endocrinology and Metabolism*.

[28] Yoshizawa F. (2014). The regulatory function of isoleucine in glucose metabolism and its clinical application. *Seikagaku*.

[29] Kuzuya M. (2013). Nutrition. *Japanese Journal of Geriatrics*.

[30] Soysal P., Isik A. T., Stubbs B. (2016). Acetylcholinesterase inhibitors are associated with weight loss in older people with dementia: a systematic review and meta-analysis. *Journal of Neurology, Neurosurgery & Psychiatry*.

[31] Spaccavento S., Del Prete M., Craca A., Fiore P. (2009). Influence of nutritional status on cognitive, functional and neuropsychiatric deficits in Alzheimer's disease. *Archives of Gerontology and Geriatrics*.

